# Clinical integration and evolution of transanal total mesorectal excision: the ideal framework in practice

**DOI:** 10.1186/1745-6215-16-S2-P8

**Published:** 2015-11-16

**Authors:** Marta Penna, Allison Hirst, Peter McCulloch, Chris Cunningham, Neil Mortensen, Roel Hompes

**Affiliations:** 1 Department of Colorectal Surgery, Churchill Hospital, Oxford, UK; 2 Nuffield Department of Surgical Sciences, Oxford, UK

## 

Transanal total mesorectal excision (TaTME) is a novel approach pioneered to tackle challenges posed by difficult pelvic dissections in colorectal surgery. As with all emerging techniques, small modifications and optimisation is often required to enhance its efficacy and safety. Designing and conducting valid scientific evaluations of new surgical procedures is difficult. The IDEAL framework (http://www.ideal-collaboration.net) provides a widely recognised structure for describing the natural evolution of surgical innovation.

## Method

We report on IDEAL Stage 2a (Prospective Development Study - PDS) development of TaTME in a tertiary centre in Oxford. We outline the stepwise evolution and modifications made on consecutive cases to improve the technique, its reproducibility and ultimately its efficacy and safety.

## Results

Since June 2013, we performed 29 TaTME procedures. The initial development involved changing to a more stable transanal platform, from glove port to Gelpoint path. By further adding an ‘Airseal’ device, better visualisation was accomplished improving stability of the pneumopelvis and smoke evacuation. Subsequently, full thickness rectotomy and dissection in a safe and efficient manner became possible. Further, the anastomotic technique was modified to ensure a more secure join. After 20 cases we adopted a synchronous approach with two teams, abdominal and perineal, leading to better operative flow and shorter operative time. All surgical techniques and peri-operative outcomes are prospectively recorded on a national registry.

## Conclusion

Describing and analysing experience of this new technique using the IDEAL PDS recommendations highlights the main learning points for surgeons. It will also contribute towards preparation for a randomized controlled trial.

